# Direction-led temporal control and expertise-based load redistribution in 45° cutting: an SPM-based analysis of ankle mechanics

**DOI:** 10.3389/fbioe.2026.1751828

**Published:** 2026-05-20

**Authors:** Xiao-shan Lei, Xiao-long Liao, Yilin Wang, Yun-chao Ma, Hao Lei

**Affiliations:** 1 Country College of P.E. and Sports, Beijing Normal University, Beijing, China; 2 Department of Physical Education, Zhongnan University of Economics and Law, Wuhan, China

**Keywords:** anklejoint stability, change of direction, injury risk, lower limb biomechanics, professional athletes, statistical parametric mapping

## Abstract

Rapid change-of-direction movements are essential in basketball and other multidirectional sports and impose substantial mechanical demands on the ankle. However, how cutting direction and athletic level jointly influence ankle-centered biomechanics across the stance phase remains insufficiently characterized. This study aimed to quantify the direction- and athletic-level-associated differences in lower-limb kinematics and kinetics during 45° cutting and to determine whether between-group effects are expressed primarily in internal joint mechanics rather than external ground reaction forces. Twenty-four healthy males (12 professional basketball athletes and 12 collegiate students) performed 45° lateral cutting (LC) and crossover cutting (CC) at a controlled approach speed (4.0 ± 0.2 m/s). Three-dimensional kinematics (250 Hz) and ground reaction forces (1,000 Hz) were synchronously recorded using motion capture and a force platform. Discrete outcomes were compared using independent-samples t-tests, and time-continuous waveforms were assessed using one-dimensional statistical parametric mapping with a mixed-design ANOVA (group × task). Cutting direction produced significant stance-phase effects across large portions of stance for ground reaction forces, ankle joint forces, and ankle joint moments, whereas ground reaction force waveforms showed no group × task interactions. Athletic-level differences were task-specific and time-localized. Professional athletes exhibited smaller non-sagittal ankle range of motion (adduction/abduction during LC; internal/external rotation during CC) and greater ankle plantarflexion moments. Significant group × task interactions were observed in frontal- and transverse-plane ankle kinematics, and the ankle adduction/abduction moment differed between groups only during CC within restricted stance intervals. These findings indicate that 45° cutting biomechanics are predominantly direction-dependent, whereas athletic level influences phase-specific modulation of ankle motion and joint moments. Athletic-level effects were primarily expressed in internal joint mechanics rather than external ground reaction forces. This information may support more targeted biomechanical assessment and training strategies for multidirectional sports.

## 1 Introduction

In basketball and similar multidirectional court sports, rapid change-of-direction (COD) maneuvers are essential for creating offensive separation and defensive adjustments, yet they are also closely linked to non-contact lower-limb injury risk. Among these maneuvers, 45° lateral cutting (LC) and 45° crossover cutting (CC) are frequently performed under high approach speeds and tight spatial constraints, generating substantial ground reaction forces and complex, sequential loading across the lower-limb kinetic chain (Sundberg et al., 2025; Singh et al., 2025). As the most distal joint interacting directly with the ground, the ankle plays a pivotal role in attenuating impact, regulating mediolateral shear, and maintaining foot–ground stability during cutting (Taylor et al., 2015; Tummala et al., 2023; Fong et al., 2007). Epidemiological evidence shows that a substantial proportion of basketball injuries stem from COD-related mechanisms (Tummala et al., 2023), with ankle sprains alone contributing 18%–23% of lower-limb injuries (Fong et al., 2007). From a pathomechanical perspective, rapid loading in the frontal and transverse planes, combined with unstable or extreme ankle postures, can increase lateral ligament strain and compromise joint stability (Zahra et al., 2024), potentially progressing to chronic ankle instability and long-term performance impairments (Kalichman et al., 2016).

To examine ankle control strategies within a single support period, we adopted a 45° cutting model that balances ecological relevance and biomechanical interpretability. Cutting maneuvers span a range of reorientation demands, and larger angles (e.g., 90° or 180°) typically require greater speed reduction, pivot-like actions, or multi-step redirection, which complicates interpretation of stance-phase ankle regulation within one contact. In contrast, a 45° cut can often be executed largely within a single stance while still imposing substantial braking and shear-control demands (Robinson and Vanrenterghem, 2012). This moderate-angle task is also relevant to ankle injury mechanisms: musculoskeletal simulations indicate that 45° cutting can increase inversion-related demands and elevate lateral ligament strain-rate requirements, aligning with mechanisms implicated in lateral ankle sprain (Jie et al., 2024; Xu et al., 2025b; Xu et al., 2025a). In parallel, computational approaches that integrate movement data with ligament mechanics models make it increasingly feasible to estimate subject-specific lateral ligament loading and fatigue-related failure risk (Xu et al., 2025a). Together, these developments motivate a time-resolved characterization of how the ankle regulates motion and internal loading across stance, particularly in the frontal and transverse planes.

Despite the ankle’s functional importance, COD biomechanics research has historically emphasized the knee and anterior cruciate ligament, while the ankle’s time-dependent kinematic–kinetic characteristics remain comparatively underexplored (Donelon et al., 2020). This gap is clinically meaningful because the ankle functions as a distal “stabilization hub,” influencing foot placement, center-of-mass control, and proximal load redistribution (Yi et al., 2024). Skill level and training background may further shape these strategies: highly trained athletes may exhibit more consistent and phase-specific regulation, whereas less trained players may display prolonged or less targeted adjustments across stance, potentially increasing cumulative exposure to destabilizing loads (Vuurberg et al., 2018; Rao et al., 2024). Importantly, many existing investigations rely primarily on discrete metrics (e.g., peak values or maximal range of motion) (Zahra et al., 2024; Yona et al., 2024), which capture only isolated instants and may obscure when during stance critical differences emerge and how load is redistributed across phases (Yona et al., 2024).

One-dimensional statistical parametric mapping (SPM) provides an effective time-continuous analytical framework for evaluating full kinematic and kinetic waveforms across stance (Yona et al., 2024). By examining entire waveform patterns (e.g., via F-statistics), SPM identifies phase-dependent divergences without reliance on arbitrary time-point selection. Prior studies demonstrate SPM’s utility in revealing task-specific coordination strategies within lower-limb biomechanics (Warmenhoven et al., 2018; Kim et al., 2024). Prior SPM studies show that interventions can alter joint moments in phase-specific manners during propulsion and landing (Huang et al., 2025), underscoring the value of waveform-based inference for COD biomechanics (Shi et al., 2025).

Therefore, the present study combined discrete-variable comparisons and statistical parametric mapping to examine lower-extremity biomechanics during 45° lateral cutting and 45° crossover cutting, with a primary focus on ankle joint kinematics and kinetics. Specifically, we compared professional basketball athletes and collegiate recreational basketball students to determine how task type modulates ankle-centered stabilization and load redistribution across the stance phase. Hip and knee variables were included as secondary outcomes to contextualize distal–proximal coordination. We hypothesized that the crossover cutting task would elicit greater frontal- and transverse-plane ankle control demands than the lateral cutting task, and that professional basketball athletes would demonstrate more phase-specific ankle regulation compared with collegiate students. Clarifying these time-dependent ankle strategies may advance understanding of cutting biomechanics and support targeted training and injury-prevention approaches for basketball populations.

## 2 Materials and methods

### 2.1 Participants

A total of 24 healthy male participants were recruited, including 12 professional basketball athletes (PA) and 12 collegiate recreational basketball students (CS). The PA group had a mean ± SD age of 20.0 ± 1.41 years, body mass of 89.3 ± 11.06 kg, height of 190.8 ± 6.39 cm, and BMI of 24.4 ± 1.96 kg/m^2^. The CS group had a mean ± SD age of 22.0 ± 1.54 years, body mass of 79.1 ± 7.62 kg, height of 183.0 ± 5.47 cm, and BMI of 23.7 ± 1.26 kg/m^2^. The PA group was recruited from the Beijing Normal University basketball team; all athletes had at least 4 years of competitive basketball experience and trained 3–4 sessions per week. The CS group was recruited from a university basketball course; all participants had more than 3 years of basketball experience and attended two basketball classes per week. This sample size is comparable to previous cutting-biomechanics studies (e.g., n = 21 females (Robbins et al., 2024); n = 17 males (Liew et al., 2020); n = 22 males (Thieschäfer et al., 2023)).

Leg dominance was determined using a standardized kicking task (kicking a ball toward a target), and all participants were right-leg dominant (Melick et al., 2017). Inclusion criteria: (1) no chronic lower-limb conditions; (2) no sports injury within the previous 6 months; (3) normal foot morphology; and (4) no clinically relevant restriction of ankle mobility. All experimental procedures were conducted at the School of Physical Education and Sports Science, Beijing Normal University. The institutional review board approved the study, and all participants provided written informed consent prior to testing.

### 2.2 Experimental procedure and data collection

To minimize the influence of footwear and apparel on biomechanical measurements, participants changed into standardized socks, shoes, and compression shorts before warm-up. Participants then began with a warm-up session and acclimatized to the laboratory environment. After warm-up, participants received standardized instruction from a coach and were required to successfully perform three supervised practice trials to ensure task familiarity.

Once participants demonstrated consistent execution, reflective markers were placed by a trained investigator to minimize placement error, and marker locations were verified by a second investigator. Markers were applied according to a modified Helen Hayes lower-limb model, including landmarks at the acromion, iliac crest, greater trochanter, femoral epicondyles, malleoli, calcaneus, and the first and fifth metatarsophalangeal joints. Three-dimensional kinematic data were recorded using an eight-camera Vicon MX-40 motion-capture system (Oxford Metrics, United Kingdom) at 250 Hz, synchronized with kinetic data from a three-dimensional force platform (9286BA, Kistler, Switzerland) sampled at 1,000 Hz. Prior to formal testing, participants completed a familiarization period to standardize performance of 45° lateral cut and 45° crossover cut maneuvers. Participants were instructed to place the foot as centrally as possible on the force platform.

**FIGURE 1:**
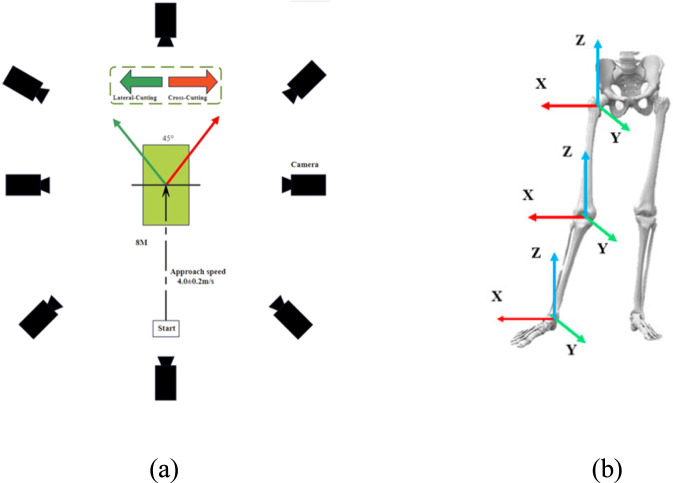
Experiment setup **(a)** and schematic diagram of the joints and coordinate systems **(b)**.

Participants approached the cutting area with an acceleration distance of at least 8 m and executed the cut with the right foot contacting the force platform. Task direction was cued by an illuminated signal on the target board, and adhesive tape on the floor indicated the required 45° cutting angle (Figure 1a). Running speed was monitored using a dual-beam timing system (Witty-Manual, Microgate, Italy) and was constrained to 4.0 ± 0.2 m/s (Liew et al., 2020). Trials were only accepted if the participant entered the cutting area within the target speed range; otherwise, the trial was repeated or excluded. This speed was selected to standardize mechanical loading and facilitate comparability with prior 45° cutting studies, and group-wise approach speeds are reported in Supplementary Tables S1–S2.

A trial was considered successful if the cut was completed within the prescribed speed range and within the taped cutting area (Figure 1). To minimize fatigue, participants rested for at least 30 s between trials. For each task condition, three valid trials were collected, and trial quality was supervised by the coach. Trials were discarded if any of the following criteria were not met: (1) complete foot contact with the force plate; (2) adherence to the prescribed running speed; or (3) absence of self-reported abnormal movement execution.

### 2.3 Biomechanical variables and data processing

Marker trajectories were labeled and gap-filled in Vicon Nexus, then exported as C3D files and processed in Visual3D (Version 5, C-Motion, Inc., Rockville, MD, United States) to compute lower-extremity kinematics and kinetics. After trajectory labeling and gap filling, no missing values remained in the exported kinematic or kinetic datasets; all participants were included in all analyses. Kinematic and kinetic waveforms were filtered using a fourth-order zero-lag Butterworth low-pass filter with cutoff frequencies of 12 Hz and 40 Hz, respectively (Liu et al., 2022; Altun et al., 2024; Xu et al., 2025a). Biomechanical variables were selected based on COD mechanisms and research focusing on athletic populations. Kinematic outcomes included hip, knee, and ankle joint angles $\left({}^{\circ}\right)$ . Kinetic outcomes included vertical ground reaction force (vGRF), hip/knee/ankle joint moments $\left(N\cdot m/\text{kg}\right)$ , and joint reaction forces $\left(N/\text{kg}\right)$ (Delp et al., 2007).

Joint kinematics and kinetics were expressed using the Visual3D joint coordinate system (JCS), following ISB recommendations and the right-hand rule (Wu et al., 2002; Moore et al., 2022). Hip, knee, and ankle joint angles and moments were defined as the motion of the distal segment relative to the proximal (reference) segment: the thigh relative to the pelvis (hip), the shank relative to the thigh (knee), and the foot relative to the shank (ankle). Joint angles and moments were resolved into flexion/extension (X component; mediolateral axis), adduction/abduction (Y component; anteroposterior axis), and internal/external rotation (Z component; vertical axis). Under the adopted Visual3D sign convention, positive values corresponded to hip flexion/adduction/internal rotation, knee extension/adduction/internal rotation, and ankle dorsiflexion/inversion/foot adduction (toe-in). Ground reaction forces and joint reaction forces were expressed in the laboratory (global) coordinate system as orthogonal components in the anteroposterior (AP), mediolateral (ML), and vertical (V) directions. Negative values indicate loading directed along the negative axis (direction) rather than a “negative magnitude.”

For clarity, component definitions are used consistently throughout the manuscript. For ground reaction forces and joint reaction forces expressed in the laboratory coordinate system, X, Y, and Z denote the mediolateral, anteroposterior, and vertical directions, respectively. For joint angles and joint moments expressed in the joint coordinate system, X, Y, and Z denote flexion–extension, adduction–abduction, and internal–external rotation, respectively.

**FIGURE 2:**
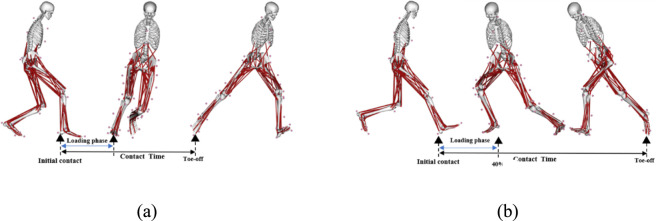
Event definition for stance-phase time normalization during the lateral **(a)** and crossover cutting tasks **(b)**.

Joint moments were normalized to body mass (Nm/kg), whereas GRFs and joint reaction forces were normalized to body weight (BW) to account for inter-individual differences in body mass. Ground reaction force thresholds were set to 20 N (Singh et al., 2025; Taylor et al., 2015). Initial contact (IC) was defined as the first frame at which vertical ground reaction force exceeded 20 N, and toe-off (TO) as the first frame after stance when vertical ground reaction force fell below 20 N. The period from IC to was defined as the stance phase (contact time) and was time-normalized to 100% of stance (Besier et al., 2001) (Figure 2). All dominant-limb biomechanical waveforms were time-normalized to 101 points (0%–100% stance) for subsequent analyses (Jlassi and Dixon, 2024).

### 2.4 Statistical analysis

An *a priori* power analysis (G*Power v3.1.9.7; mixed-design ANOVA; f = 0.25; α = 0.05; power = 0.80) indicated a minimum sample size of 22; therefore, 24 participants were recruited. Statistical analyses were performed using IBM SPSS Statistics 22.0 (SPSS Corp., United States) for discrete variables and MATLAB (R2020a; The MathWorks, United States) with SPM1D v0.4 (www.spm1d.org) for time-series analyses. The significance level was set at α = 0.05. For each cutting task (lateral cut task and crossover cut task), between-group differences (professional basketball athletes vs. collegiate students) in time-discrete lower-extremity variables were examined using independent-samples t-tests. Discrete outcomes included hip, knee, and ankle joint-angle ROM in flexion/extension, adduction/abduction, and internal/external rotation, as well as peak joint reaction forces and peak joint moments. When the assumption of homogeneity of variance was violated, Welch’s t-test was applied. Effect sizes were calculated using Cohen’s d.

Time-continuous data were analyzed using one-dimensional statistical parametric mapping (SPM1D). A two-way mixed-design ANOVA was conducted with Group (professional athletes vs. collegiate students) as the between-subject factor and Task (lateral cut vs. crossover cut) as the within-subject factor. Statistical inference was based on random field theory, which controls the family-wise error rate across the stance-phase continuum (α = 0.05). When a significant main effect or interaction was detected, planned *post hoc* SPM{t} tests were performed to examine simple effects. Four *a priori* contrasts were evaluated: (1) athletes vs. students during the lateral cut, (2) athletes vs. students during the crossover cut, (3) crossover vs. lateral cut within athletes, and (4) crossover vs. lateral cut within students. Between-group contrasts were assessed using two-sample SPM{t} tests, whereas within-group contrasts were assessed using paired SPM{t} tests. To control the family-wise error rate across these four planned contrasts, the Holm–Bonferroni procedure was applied within each outcome family. Outcome variables were organized into predefined families based on biomechanical constructs: ground reaction forces (GRF family), ankle joint angles (angle family), ankle joint reaction forces (force family), ankle joint moments (moment family), and secondary hip and knee variables (secondary family). No additional multiplicity correction was applied across outcome families because these families represent distinct biomechanical domains and address different research questions. Therefore, findings from secondary families were interpreted as exploratory. Significant SPM effects were reported as suprathreshold clusters with cluster-level p-values and corresponding time intervals (% stance). Primary outcomes were defined *a priori* as ankle joint kinematics and kinetics, whereas hip and knee variables were considered secondary outcomes.

## 3 Results

### 3.1 Inter-group comparisons based on discrete variables

Approach speed did not differ between groups (see Supplementary Tables S1–S2). During the lateral cutting task (Table 1), several between-group differences were observed. Compared with collegiate students, professional athletes exhibited a smaller ankle joint-angle ROM in ankle adduction/abduction (Y component) (23.44 ± 4.30 vs. 26.57 ± 6.64; p = 0.031), suggesting reduced adduction/abduction excursion under the adopted coordinate convention. Professional athletes also demonstrated a lower peak knee joint reaction force in the mediolateral (X) component (1.68 ± 0.95 vs. 2.49 ± 1.21; p = 0.004). In addition, professional athletes showed greater compressive knee joint reaction force (−24.76 ± 5.65 vs. −20.42 ± 5.52 BW; p = 0.003), indicating higher loading along the negative Z-axis under our sign convention. At the ankle, professional athletes further exhibited a more negative peak joint reaction force in the anteroposterior (Y) component (−19.50 ± 3.31 vs. −16.10 ± 4.55; p = 0.007) and a more negative peak ankle flexion/extension (plantarflexion/dorsiflexion; X-component) moment (−2.23 ± 0.59 vs. −1.95 ± 0.38; p = 0.023). Collectively, these findings are consistent with task- and component-specific differences in joint loading and kinematics between athletic levels during lateral cutting.

**TABLE 1:** Inter-group comparison of lower-limb joint angles, joint reaction forces, and peak joint moments during the LC task.

Joint	Parameters	Axial direction	PA group (mean, SD)	CS group (mean, SD)	p value
Hip	ROM of joint angles $\left({}^{\circ}\right)$	X	54.16±12.07	53.77±7.98	0.878
		Y	18.48±7.84	18.66±4.69	0.730
		Z	19.71±7.55	15.81±6.27	0.187
	Peak joint force (BW)	X	4.19±3.64	4.06±4.37	0.894
		Y	−14.92±4.93	−16.31 ± 6.15	0.324
		Z	−20.30 ± 4.54	−18.79 ± 3.76	0.147
	Peak joint moment $\left(N\cdot m/\text{kg}\right)$	X	−4.39 ± 2.31	−4.49 ± 2.43	0.870
		Y	−1.76 ± 0.72	−1.83 ± 1.28	0.791
		Z	−1.59 ± 1.11	−1.65 ± 1.10	0.849
Knee	ROM of joint angles $\left({}^{\circ}\right)$	X	46.81 ± 12.51	45.69 ± 12.13	0.650
		Y	8.67 ± 3.09	9.62 ± 4.32	0.318
		Z	13.73 ± 5.67	11.16 ± 4.48	0.252
	Peak joint force (BW)	X	1.68 ± 0.95	2.49 ± 1.21	0.004**
		Y	11.00 ± 1.95	11.45 ± 1.77	0.331
		Z	−24.76 ± 5.65	−20.42 ± 5.52	0.003**
	Peak joint moment $\left(N\cdot m/\text{kg}\right)$	X	3.38 ± 1.03	3.65 ± 0.56	0.186
		Y	0.79 ± 0.44	0.75 ± 0.57	0.821
		Z	0.15 ± 0.12	0.18 ± 0.11	0.316
Ankle	ROM of joint angles $\left({}^{\circ}\right)$	X	58.85 ± 9.96	59.15 ± 11.11	0.912
		Y	23.44 ± 4.30	26.57 ± 6.64	0.031*
		Z	24.82 ± 6.01	23.80 ± 10.99	0.359
	Peak joint force (BW)	X	12.02 ± 4.46	11.13 ± 3.83	0.389
		Y	−19.50 ± 3.31	−16.10 ± 4.55	0.007**
		Z	−15.47 ± 6.26	−14.66 ± 2.53	0.508
	Peak joint moment $\left(N\cdot m/\text{kg}\right)$	X	−2.23 ± 0.59	−1.95 ± 0.38	0.023*
		Y	−1.94 ± 0.55	−1.87 ± 0.36	0.547
		Z	0.93±0.37	0.75±0.39	0.069

PA, professional athlete group; CS, college student group; LC, lateral cutting task; BW, Expressed as multiples of body weight. *p<0.05,**p<0.01 .

In the crossover cut task (Table 2), professional athletes exhibited a greater peak hip adduction/abduction (Y-component) moment (1.13 ± 0.61 vs. 0.64 ± 0.73; p = 0.008). At the knee, professional athletes showed a smaller peak knee internal/external rotation (Z-component) moment (0.21 ± 0.07 vs. 0.26 ± 0.11; p = 0.034). At the ankle, professional athletes demonstrated a more negative peak ankle joint reaction force in the mediolateral (X) component (−2.37 ± 1.71 vs. −0.62 ± 2.05; p = 0.002) and a more negative peak ankle flexion/extension (plantarflexion/dorsiflexion; X-component) moment (−2.45 ± 0.44 vs. −2.00 ± 0.57; p = 0.001). Collectively, these discrete-variable findings suggest task- and component-specific differences in joint loading between athletic levels during crossover cutting, which motivated the subsequent SPM analyses to determine the stance-phase intervals over which group differences occurred.

**TABLE 2:** Inter-group comparison of lower-limb joint angles, joint reaction forces, and peak joint moments between PA and CS during the CC task.

Joint	Parameters	Axial direction	PA group (mean, SD)	CS group (mean, SD)	p value
Hip	ROM of joint angles $\left({}^{\circ}\right)$	X	65.52±14.12	62.49±13.15	0.411
		Y	18.74 ± 8.16	15.53 ± 5.37	0.287
		Z	22.04 ± 8.52	19.94 ± 8.38	0.699
	Peak joint force (BW)	X	3.79 ± 3.27	2.94 ± 1.54	0.243
		Y	−1.16 ± 5.50	−1.19 ± 4.12	0.520
		Z	−13.43 ± 5.60	−11.73 ± 2.13	0.208
	Peak joint moment $\left(N\cdot m/\text{kg}\right)$	X	−2.80 ± 1.60	−2.99 ± 1.98	0.690
		Y	1.13 ± 0.61	0.64 ± 0.73	0.008**
		Z	−0.81 ± 0.55	−0.61 ± 0.49	0.153
Knee	ROM of joint angles $\left({}^{\circ}\right)$	X	59.17 ± 12.82	60.56 ± 14.69	0.836
		Y	10.41 ± 3.47	11.91 ± 4.56	0.171
		Z	10.14 ± 5.86	11.04 ± 5.30	0.711
	Peak joint force $\left(\text{BW}\right)$	X	2.07 ± 1.32	2.68 ± 1.50	0.152
		Y	6.92 ± 1.98	7.84 ± 1.66	0.093
		Z	−15.22 ± 9.02	−13.02 ± 3.27	0.308
	Peak joint moment $\left(N\cdot m/\text{kg}\right)$	X	2.87 ± 1.02	2.47 ± 1.17	0.173
		Y	0.75 ± 0.38	0.74 ± 0.37	0.965
		Z	0.21 ± 0.07	0.26 ± 0.11	0.034*
Ankle	ROM of joint angles $\left({}^{\circ}\right)$	X	54.55 ± 10.91	57.73 ± 10.86	0.418
		Y	19.82 ± 7.58	18.71 ± 6.98	0.974
		Z	19.05 ± 7.30	22.74 ± 6.20	0.047
	Peak joint force $\left(\text{BW}\right)$	X	−2.37 ± 1.71	−0.62±2.05	0.002**
		Y	−14.14 ± 4.98	−13.30 ± 2.97	0.474
		Z	−10.89±7.98	−8.93±3.16	0.252
	Peak joint moment $\left(N\cdot m/\text{kg}\right)$	X	−2.45±0.44	−2.00±0.57	0.001**
		Y	0.30±0.23	0.40±0.19	0.077
		Z	−0.22±0.21	−0.25±0.27	0.688

PA, professional athlete group; CS, college student group; CC, crossover cutting task; BW, Expressed as multiples of body weight. *p<0.05,**p<0.01 .

### 3.2 Ground reaction force

**FIGURE 3:**
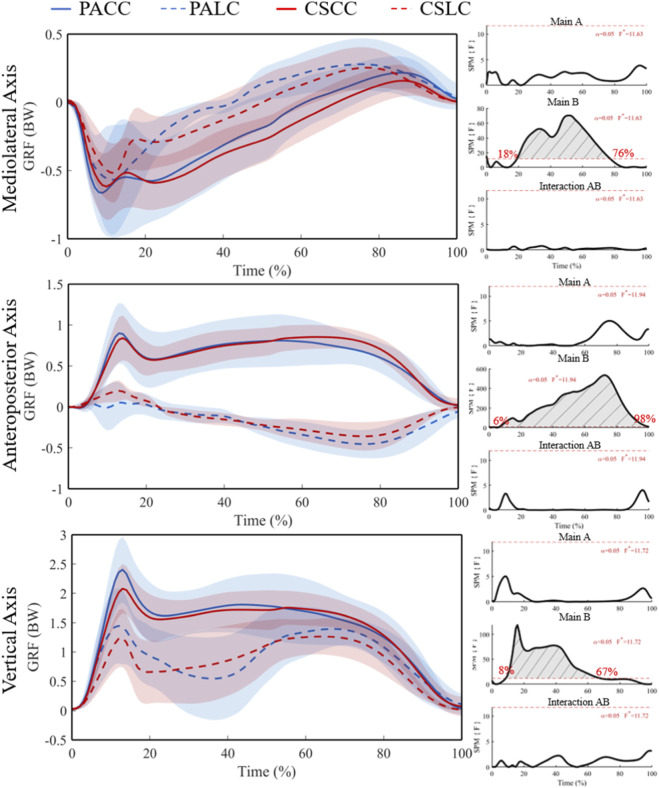
Mean (SD) ground reaction force waveforms (BW) across stance (0%–100%) with corresponding SPM{F} trajectories from the 2 × 2 mixed ANOVA. Panels show the Group main effect (A), Task main effect (B), and Group × Task interaction (AB). The horizontal red dashed line indicates the critical threshold; grey shaded regions denote suprathreshold clusters. Abbreviations: PA, professional athletes; CS, collegiate students; LC, lateral cut; CC, crossover cut; PALC/PACC/CSLC/CSCC denote group-by-task conditions.

**FIGURE 4:**
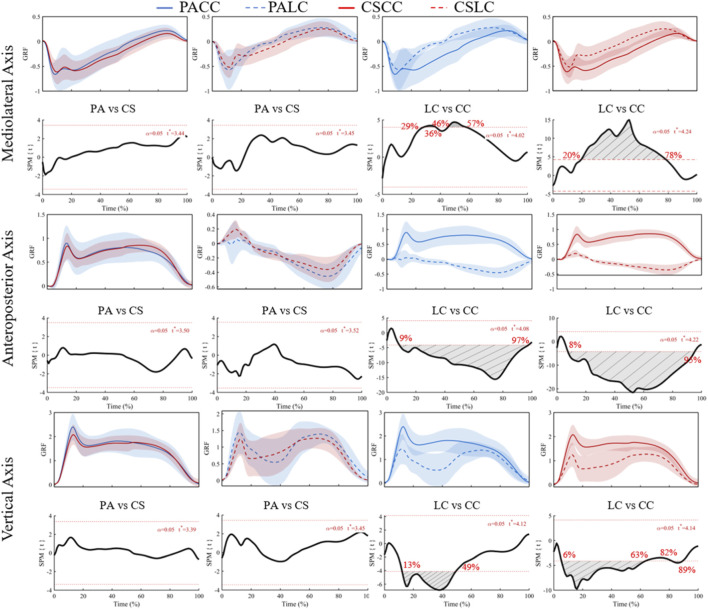
Mean (SD) ground reaction forces (BW) across stance (0%–100%) and corresponding SPM{t} trajectories for planned *post hoc* comparisons. PA (blue) and CS (red) groups; LC (solid) and CC (dashed) conditions. Red dashed line represents the critical threshold. Grey dashed-line area represents the supra-threshold cluster region. Abbreviations: PA, professional athletes; CS, collegiate students; LC, lateral cut; CC, crossover cut; PALC/PACC/CSLC/CSCC denote group-by-task conditions.

Ground reaction forces were evaluated as mediolateral (X), anteroposterior (Y), and vertical (Z) components across stance (Figures 3, 4). For the mediolateral component of the ground reaction force, SPM ANOVA revealed no significant main effect of group and no significant group × task interaction, but a significant main effect of task at 0%–0.6% and 18.1%–76.4% of stance (F* = 10.71; Figure 3). Post hoc SPM{t} tests showed no significant between-group differences during either the lateral cut task (t* = 3.44) or the crossover cut task (t* = 3.45) (Figure 4). Significant within-group task effects were detected in both groups: 29%–36% and 46%–57% of stance in professional basketball athletes (t* = 4.02) and 20%–78% in collegiate students (t* = 4.24) (Figure 4).

For the anteroposterior component of the ground reaction force, a significant main effect of task was detected at 0%–0.9% and 5.7%–98.3% of stance (F* = 11.94; Figure 3), with no significant main effect of group or interaction. Post hoc analyses indicated significant within-group task differences spanning 9%–97% in professional basketball athletes (t* = 4.08) and 8%–95% in collegiate students (t* = 4.22) (Figure 4). Between-group comparisons were not significant during both tasks (t* = 3.50 during the lateral cut task; t* = 3.52 during the crossover cut task).

For the vertical component of the ground reaction force, a significant main effect of task was observed from 7.6% to 66.9% of stance (F* = 11.72; Figure 3), with no significant group or interaction effects. Post hoc analyses identified significant within-group task differences at 13%–49% in professional basketball athletes (t* = 4.12) and at 6%–63% plus 82%–89% in collegiate students (t* = 4.14) (Figure 4). Between-group comparisons were not significant during both tasks (t* = 3.39 during the lateral cut task; t* = 3.45 during the crossover cut task). Overall, GRF waveform differences were primarily task-dependent (lateral vs. crossover), whereas the absence of a significant group × task interaction indicates that task-related GRF changes were broadly comparable between groups under the current sample size.

### 3.3 Ankle joint angle

**FIGURE 5:**
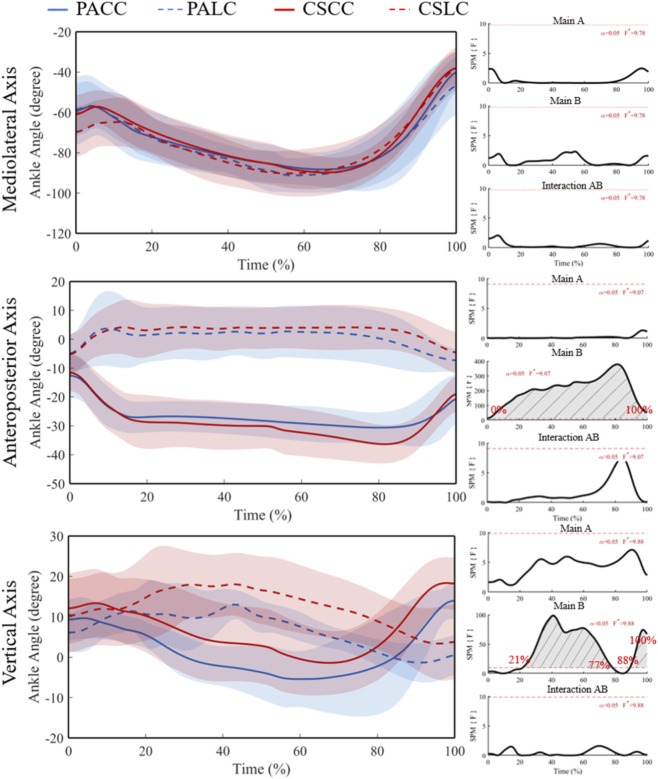
Mean (SD) ankle joint angle (°) across stance (0%–100%) with corresponding SPM{F} trajectories from the 2 × 2 mixed ANOVA. Panels show the Group main effect (A), Task main effect (B), and Group × Task interaction (AB). The horizontal red dashed line indicates the critical threshold; grey shaded regions denote suprathreshold clusters. Abbreviations: PA, professional athletes; CS, collegiate students; LC, lateral cut; CC, crossover cut; PALC/PACC/CSLC/CSCC denote group-by-task conditions.

**FIGURE 6:**
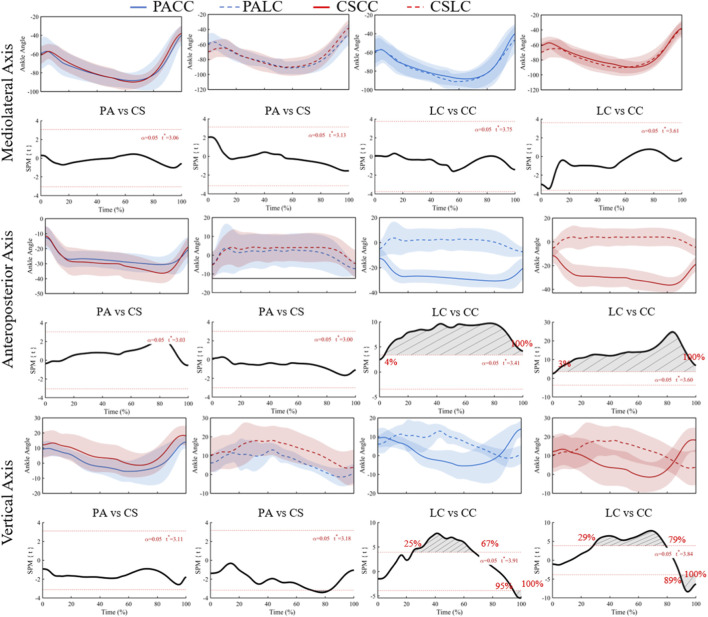
Mean (SD) patterns for the ankle joint angle (°) across stance (0%–100%) and corresponding SPM{t} trajectories. PA (blue) and CS (red) groups; LC (solid) and CC (dashed) conditions. Red dashed line represents the critical threshold. Grey dashed-line area represents the supra-threshold cluster region. Abbreviations: PA, professional athletes; CS, collegiate students; LC, lateral cut; CC, crossover cut; PALC/PACC/CSLC/CSCC denote group-by-task conditions.

No significant effects were detected for the ankle flexion/extension angle (X component) (Figure 5; F* = 9.78), and all *post hoc* comparisons were not significant (Figure 6). For the ankle adduction/abduction angle (Y component), ANOVA revealed a significant group × task interaction spanning 0%–100% of stance (F* = 9.07; Figure 5), with no significant main effects. Post hoc tests showed significant within-group task differences from 4% to 100% in professional basketball athletes (t* = 3.41) and from 3% to 100% in collegiate students (t* = 3.60) (Figure 6), while between-group comparisons were not significant during both tasks (t* = 3.03 during the lateral cut task; t* = 3.33 during the crossover cut task).

For the ankle internal/external rotation angle (Z component), a significant group × task interaction was observed at 21%–77% and 88%–100% of stance (F* = 9.88; Figure 5). Post hoc analyses identified significant within-group task differences at 25%–67% and 95%–100% in professional basketball athletes (t* = 3.91) and at 29%–79% plus 89%–100% in collegiate students (t* = 3.84) (Figure 6), with between-group comparisons not significant during both tasks.

### 3.4 Ankle joint forces

**FIGURE 7:**
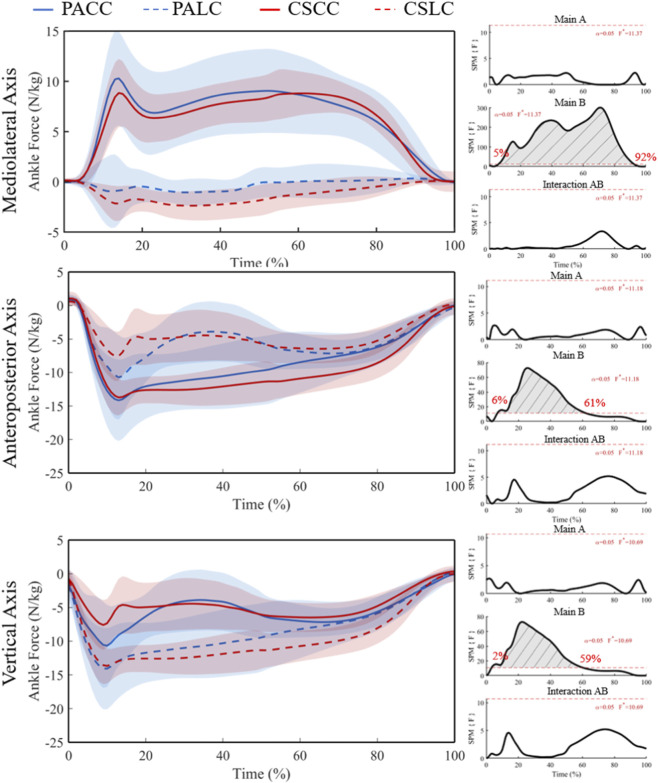
Mean (SD) ankle joint force (N/kg) across stance (0%–100%) with corresponding SPM{F} trajectories from the 2 × 2 mixed ANOVA. Panels show the Group main effect (A), Task main effect (B), and Group × Task interaction (AB). The horizontal red dashed line indicates the critical threshold; grey shaded regions denote suprathreshold clusters. Abbreviations: PA, professional athletes; CS, collegiate students; LC, lateral cut; CC, crossover cut; PALC/PACC/CSLC/CSCC denote group-by-task conditions.

**FIGURE 8:**
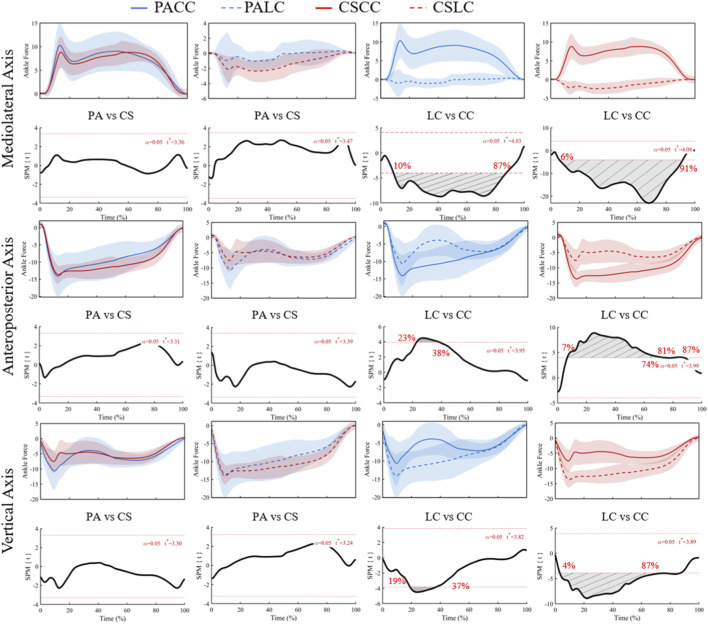
Mean (SD) patterns for the ankle joint force (BW) across stance (0%–100%) and corresponding SPM{t} trajectories. PA (blue) and CS (red) groups; LC (solid) and CC (dashed) conditions. Red dashed line represents the critical threshold. Grey dashed-line area represents the supra-threshold cluster region. Abbreviations: PA, professional athletes; CS, collegiate students; LC, lateral cut; CC, crossover cut; PALC/PACC/CSLC/CSCC denote group-by-task conditions.

For ankle joint force in the mediolateral (X) component, ANOVA revealed a significant main effect of task from 5% to 91% of stance (F* = 11.37; Figure 7), with no significant main effect of group or interaction. Post hoc tests showed no significant between-group differences during either task (t* = 3.36 during the lateral cut task; t* = 3.47 during the crossover cut task), but significant within-group task differences from 10% to 87% in professional basketball athletes (t* = 4.03) and 6%–91% in collegiate students (t* = 4.08) (Figure 8).

For ankle joint force in the anteroposterior (Y) component, a significant main effect of task was detected from 6% to 61% of stance (F* = 11.18; Figure 7), with no significant group or interaction effects. Between-group comparisons were not significant during both tasks (t* = 3.31 during the lateral cut task; t* = 3.39 during the crossover cut task). Within-group task differences were significant at 23%–38% in professional basketball athletes (t* = 3.95) and at 7%–74% plus 81%–87% in collegiate students (t* = 3.99) (Figure 8).

For ankle joint force in the vertical (Z) component, ANOVA revealed a significant main effect of task from 2% to 59% of stance (F* = 10.69; Figure 7), with no significant group or interaction effects. Between-group comparisons were not significant (t* = 3.30 during the lateral cut task; t* = 3.24 during the crossover cut task). Within-group task differences were significant at 19%–37% in professional basketball athletes (t* = 3.82) and at 4%–87% in collegiate students (t* = 3.89) (Figure 8).

### 3.5 Ankle joint moment

**FIGURE 9:**
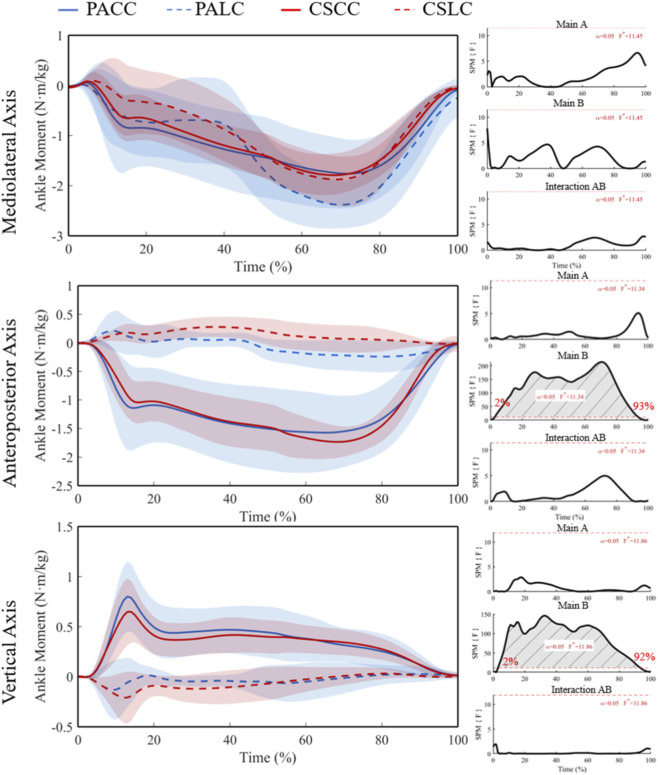
Mean (SD) ankle joint moment (Nm/kg) across stance (0%–100%) with corresponding SPM{F} trajectories from the 2 × 2 mixed ANOVA. Panels show the Group main effect (A), Task main effect (B), and Group × Task interaction (AB). The horizontal red dashed line indicates the critical threshold; grey shaded regions denote suprathreshold clusters. Abbreviations: PA, professional athletes; CS, collegiate students; LC, lateral cut; CC, crossover cut; PALC/PACC/CSLC/CSCC denote group-by-task conditions.

**FIGURE 10:**
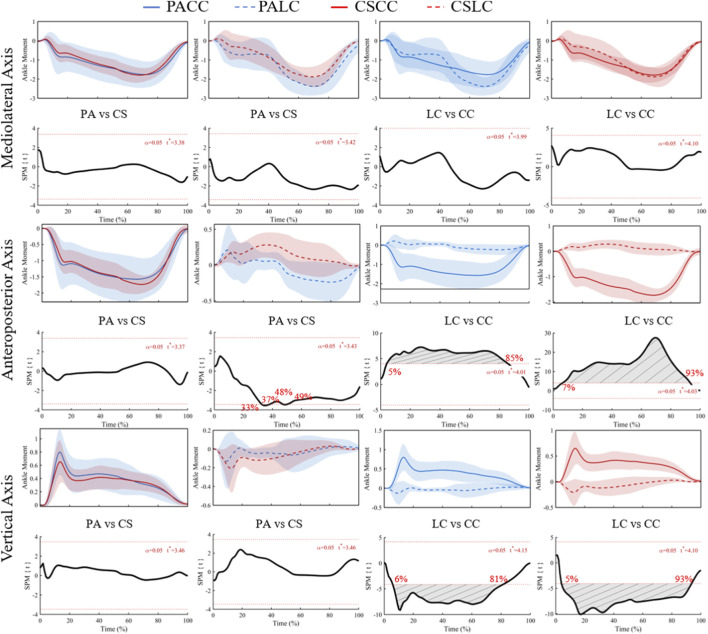
Mean (SD) patterns for the ankle joint moment (Nm/kg) across stance (0%–100%) and corresponding SPM{t} trajectories. PA (blue) and CS (red) groups; LC (solid) and CC (dashed) conditions. Red dashed line represents the critical threshold. Grey dashed-line area represents the supra-threshold cluster region. Abbreviations: PA, professional athletes; CS, collegiate students; LC, lateral cut; CC, crossover cut; PALC/PACC/CSLC/CSCC denote group-by-task conditions.

No significant effects were detected for the ankle flexion/extension (plantarflexion/dorsiflexion; X-component) moment (Figure 9; F* = 11.45), and all *post hoc* comparisons were not significant (Figure 10). For the ankle adduction/abduction (Y-component) moment, ANOVA identified a significant main effect of task from 2% to 90% of stance (F* = 11.34; Figure 9), with no significant interaction. Post hoc analyses showed no significant between-group differences during the lateral cut task (t* = 3.37), but significant between-group differences during the crossover cut task at 33%–37% and 48%–49% (t* = 3.43) (Figure 10). Within-group task differences were significant from 35% to 85% in professional basketball athletes (t* = 4.01) and 7%–93% in collegiate students (t* = 4.03) (Figure 10).

For the ankle internal/external rotation (Z-component) moment, ANOVA revealed a significant main effect of task from 2% to 92% of stance (F* = 11.86; Figure 9), with no significant interaction. Between-group comparisons were not significant during both tasks (t* = 3.46 during the lateral cut task; t* = 3.46 during the crossover cut task). Within-group task differences were significant from 6% to 81% in professional basketball athletes (t* = 4.15) and 5%–93% in collegiate students (t* = 4.10) (Figure 10).

## 4 Discussion

This study aimed to clarify how cutting direction (45° lateral cut vs. 45° crossover cut) and athletic level shape ankle biomechanics and lower-limb joint loading during stance. The principal findings were that cutting direction accounted for the largest time-dependent differences in biomechanical waveforms in GRF and ankle kinetics, whereas athletic between-group differences associated with athletic level were more time-localized and expressed primarily in non-sagittal ankle kinematics and selected joint loading features. Importantly, because we did not directly quantify physiological fitness or strength, these effects should be interpreted as athletic-level-associated rather than as “pure expertise” effects.

A key observation was that GRF waveforms were largely governed by task direction, with limited evidence of group effects. Under standardized task constraints, this pattern suggests that both groups were exposed to broadly similar external demands, and that between-group differences associated with athletic level may lie less in the magnitude of external loading and more in how joint-level mechanics manage and distribute that load across stance. This interpretation aligns with COD biomechanics emphasizing that direction changes primarily influence braking–propulsion requirements and mediolateral stabilization demands, thereby shaping force application over time (Dos’Santos et al., 2021; Singh et al., 2025).

Direction-specific modulation was particularly evident in ankle kinematics, including group × task interactions in ankle adduction/abduction (Y-component) and internal/external rotation (Z-component) angles. These non-sagittal planes are biomechanically salient because cutting requires resisting shear and maintaining foot–ankle stability while the center of mass is redirected. Crossover cutting, which typically entails more cross-body transition, is expected to challenge transverse and frontal stability to a greater extent than lateral cutting, and the present results support that the ankle’s multiplanar motion pattern is sensitive to this COD context (Schreurs et al., 2017; Donelon et al., 2020). Importantly, the interaction effects indicate that athletic-level-associated differences do not reflect a uniform shift in ankle motion; rather, cutting direction alters which rotational component is most discriminative between groups.

The discrete-variable findings further indicated task-specific differences in ankle ROM and moments between groups. Professionals demonstrated reduced ankle adduction/abduction ROM (Y component) during lateral cutting and reduced ankle internal/external rotation ROM (Z component) during crossover cutting, alongside larger ankle plantarflexion/dorsiflexion moments (flexion/extension; X component). These outcomes can be interpreted objectively as a pattern of reduced non-sagittal excursion paired with greater ankle plantarflexion during key phases of the stance. Notably, without direct measures of EMG or muscle activation, these results should not be taken as evidence of specific neuromuscular “synergy” strategies; a parsimonious explanation is that this pattern may reflect greater plantarflexor moment capacity or different joint stiffness regulation, enabling them to generate higher plantarflexion moments while limiting excessive non-sagittal excursions under comparable external demands (Liew et al., 2020). Besides, constraining non-sagittal ROM may reduce time spent near injury-prone positions, whereas higher plantarflexion moments can support efficient transition from braking to propulsion—both consistent with performance-oriented COD demands (Dos’Santos et al., 2021).

Waveform-level analyses reinforced that task direction broadly drove ankle kinetic differences, while between-group differences associated with athletic level were restricted to specific intervals, including differences in the ankle adduction/abduction (Y-component) moment during crossover cutting. This phase specificity matters because injury-relevant joint loading is time dependent, with potentially critical loading occurring during early-to-mid stance when the foot is stabilizing under shear (Fong et al., 2007; Vuurberg et al., 2018). The present data therefore support a conservative, measurement-grounded interpretation: Crossover cutting places higher requirements on frontal-plane ankle moment generation (adduction/abduction, i.e., inversion–eversion depending on sign convention) during specific stance intervals in certain stance windows, and professionals demonstrate a different magnitude profile during those windows.

Beyond the ankle, professionals exhibited larger hip adduction/abduction (Y-component) moments and smaller knee internal/external rotation (Z-component) moments during crossover cutting. This proximal pattern can be interpreted as an objective redistribution of joint loading across the proximal–distal chain during cross-body redirection; however, because performance outcomes and injury-related endpoints were not assessed, the functional implications of this redistribution cannot be determined from the present data (Donelon et al., 2020). Such a redistribution is consistent with COD literature emphasizing that hip and trunk control can influence distal mechanics and knee loading, particularly when cutting direction increases frontal/transverse demands (Dos’Santos et al., 2021).

The results also inform more concrete, data-aligned training implications. Because athletic-level-associated differences were most evident in non-sagittal ankle motion and in phase-localized mediolateral component ankle adduction/abduction (Y-component) moments during crossover cutting, training should explicitly target these measurable features: Mediolateral component ankle stabilization and inversion–eversion control, especially relevant for crossover cutting where mediolateral component moment differences were observed (e.g., single-leg balance with external perturbations; lateral hop-to-cut with stable foot placement; resisted eversion/inversion strengthening targeting peroneal control) (Vuurberg et al., 2018). Transverse-plane control under direction-specific constraints, particularly for crossover tasks where transverse ROM discrimination was seen (e.g., planned-to-unplanned crossover cuts with cue-based direction change; drills emphasizing controlled foot progression angle and trunk alignment) (Singh et al., 2025). Proximal mediolateral component strength and control (hip abductors/adductors and trunk lateral control) to support mediolateral stability and potentially reduce compensatory distal excursions during crossover cutting.

Several limitations should be acknowledged. First, force-plate targeting and any constrained foot placement may alter natural COD mechanics by changing step length, foot progression angle, or trunk positioning; future designs using larger instrumented surfaces or multi-plate walkways can reduce targeting effects. Second, only three successful trials per condition were collected; future work may consider additional trials to improve reliability. Third, generalizability is limited to young healthy males, and findings may differ in females, youth athletes, or individuals with prior ankle sprain history. Finally, electromyographic data were not collected, limiting interpretation to kinematics and kinetics rather than muscle activation. Future studies integrating EMG, unanticipated cutting, fatigue protocols, and larger cohorts will be important to test whether the observed phase-localized kinetic differences translate to performance or injury-relevant outcomes. Accordingly, recreational or developing athletes should train crossover-specific ankle control in adduction/abduction (Y) and internal/external rotation (Z), together with plantarflexor moment capacity (flexion/extension; X).

## 5 Conclusion

This study investigated how cutting direction (45° lateral cut vs. 45° crossover cut) and athletic level (professional basketball athletes vs. collegiate recreational students) shape multiplanar ankle biomechanics and lower-limb joint loading during stance. Cutting direction (45° lateral vs. crossover) accounted for the most pronounced stance-phase differences in GRF and ankle kinetics. Athletic level was associated with smaller, task-specific, and phase-localized differences, primarily in non-sagittal ankle kinematics—ankle adduction/abduction (Y component) and internal/external rotation (Z component)—and in the ankle adduction/abduction (Y-component) moment profile during crossover cutting, together with distinct proximal joint moment characteristics. Overall, the mechanical demands of 45° cutting were driven mainly by the redirection task, whereas athletic level influenced how joint loading was distributed and timed across the lower limb. Training should target crossover-specific ankle control in the Y- and Z-component rotations and plantarflexor (X-component) moment capacity to meet phase-localized stance demands.

## Data Availability

The original contributions presented in the study are included in the article/Supplementary Material, further inquiries can be directed to the corresponding author.
