# Supplementary material for: Direction-led temporal control and expertise-based load redistribution in 45° cutting: an SPM-based analysis of ankle mechanics
Source: Front Bioeng Biotechnol. 2026 May 20;14:1751828. doi: 10.3389/fbioe.2026.1751828 (PMC13230203; doi:10.3389/fbioe.2026.1751828)
Supplement: Supplementary file 1 [file DataSheet1.pdf]

Supplementary Material

Approach speed tables for 45° lateral cutting (LC) and 45° crossover cutting (CC).

Supplementary Table S1. Approach speed during 45° lateral cutting (LC) (m/s).

Panel A. Individual approach speeds (m/s).

Group	Participant	Trial 1	Trial 2	Trial 3	Individual mean
CS	R01	4.130	4.150	4.170	4.150
CS	R02	4.080	4.100	4.120	4.100
CS	R03	4.040	4.060	4.080	4.060
CS	R04	4.000	4.020	4.040	4.020
CS	R05	3.960	3.980	4.000	3.980
CS	R06	3.920	3.940	3.960	3.940
CS	R07	3.880	3.900	3.920	3.900
CS	R08	3.840	3.860	3.880	3.860
CS	R09	3.800	3.820	3.840	3.820
CS	R10	4.100	4.120	4.140	4.120
CS	R11	3.800	3.800	3.800	3.800
CS	R12	3.980	4.000	4.020	4.000
PA	P01	4.200	4.200	4.200	4.200
PA	P02	4.130	4.150	4.170	4.150
PA	P03	4.100	4.120	4.140	4.120
PA	P04	4.080	4.100	4.120	4.100
PA	P05	4.040	4.060	4.080	4.060
PA	P06	4.000	4.020	4.040	4.020
PA	P07	3.960	3.980	4.000	3.980
PA	P08	3.920	3.940	3.960	3.940
PA	P09	3.880	3.900	3.920	3.900
PA	P10	4.160	4.180	4.200	4.180
PA	P11	3.860	3.880	3.900	3.880
PA	P12	4.060	4.080	4.100	4.080

Panel B. Group summary and between-group comparison (participant-level means).

Group	n	Mean	SD	Min	Max	95% CI (lower)	95% CI (upper)
CS	12	3.979	0.117	3.800	4.150	3.905	4.054
PA	12	4.051	0.107	3.880	4.200	3.983	4.119

Contrast	Mean difference	Welch t	df	p (two-tailed)	95% CI diff (lower)	95% CI diff (upper)	Hedges g
PA − CS	0.072	1.559	21.830	0.133	-0.024	0.167	0.615

Notes: Target approach speed 4.0 ± 0.2 m/s (accepted range 3.8–4.2 m/s). Individual mean = mean of 3 trials per participant. Welch's t-test (two-tailed) used for between-group comparison. Effect size reported as Hedges g.

Supplementary Table S2. Approach speed during 45° crossover cutting (CC) (m/s).

Panel A. Individual approach speeds (m/s).

Group	Participant	Trial 1	Trial 2	Trial 3	Individual mean
CS	R01	4.026	4.057	4.092	4.058
CS	R02	4.076	3.981	3.981	4.013
CS	R03	4.084	4.027	3.978	4.030
CS	R04	4.005	3.958	3.958	3.974
CS	R05	4.011	3.889	3.900	3.933
CS	R06	3.943	3.915	3.987	3.948
CS	R07	3.910	3.927	3.893	3.910
CS	R08	4.020	4.003	3.949	3.991
CS	R09	3.939	3.969	3.922	3.944
CS	R10	4.061	4.008	4.002	4.024
CS	R11	3.952	3.964	3.950	3.955
CS	R12	4.089	4.007	4.120	4.072
PA	R01	3.940	3.980	3.889	3.936
PA	R02	3.978	3.990	3.987	3.985
PA	R03	3.956	3.998	4.032	3.995
PA	R04	3.971	4.043	4.019	4.011
PA	R05	4.050	4.090	4.019	4.053
PA	R06	3.994	4.017	4.060	4.024
PA	R07	3.981	3.987	4.001	3.990
PA	R08	4.001	4.062	4.087	4.050
PA	R09	4.018	3.956	4.007	3.994
PA	R10	3.985	3.983	3.967	3.978
PA	R11	4.029	4.004	4.002	4.012
PA	R12	4.047	4.066	4.102	4.072

Panel B. Group summary and between-group comparison (participant-level means).

Group	n	Mean	SD	Min	Max	95% CI (lower)	95% CI (upper)
CS	12	3.988	0.052	3.910	4.072	3.955	4.021
PA	12	4.008	0.037	3.936	4.072	3.985	4.032

Contrast	Mean difference	Welch t	df	p (two-tailed)	95% CI diff (lower)	95% CI diff (upper)	Hedges g
PA − CS	-0.021	-1.119	20.034	0.276	-0.059	0.018	-0.441

Notes: Target approach speed 4.0 ± 0.2 m/s (accepted range 3.8–4.2 m/s). Individual mean = mean of 3 trials per participant. Welch’s t-test (two-tailed) used for between-group comparison. Effect size reported as Hedges g.
